# ‘There was a pivotal moment’. The dynamics, transitions, adaptations and trajectories of nursing at the front-line in the UK during the COVID-19 pandemic

**DOI:** 10.1371/journal.pone.0295394

**Published:** 2024-02-29

**Authors:** Anna Rachel Conolly, Jill Maben, Ruth Abrams, Ruth Harris, Daniel Kelly, Bridie Kent, Keith Couper, Emma Rowland

**Affiliations:** 1 School of Health Sciences, University of Surrey, Guildford, United Kingdom; 2 Florence Nightingale Faculty of Nursing, Midwifery & Palliative Care, King’s College London, London, United Kingdom; 3 School of Nursing, Cardiff University, Cardiff, United Kingdom; 4 School of Nursing and Midwifery, University of Plymouth, Plymouth, United Kingdom; 5 Warwick Medical School, University of Warwick and University Hospitals Birmingham NHS Foundation Trust, Warwick, United Kingdom; University of Strathclyde Faculty of Humanities and Social Sciences, UNITED KINGDOM

## Abstract

Using qualitative interview data (n = 142 interviews) generated with 50 nurses, over the course of the COVID-19 pandemic, this paper traces the trajectories of nurses in the UK and attempts to unpick the interplay between structure and agency in their narratives. Interviews were inductively analysed for themes and an additional narrative analysis was undertaken to preserve the form of each participant’s narrative. We argue that nurses’ pandemic trajectories occurred within the ‘psychological vulnerability-stigma nexus’ which operates within health and social care providers in the UK and whilst constraining nurses’ agency at times it could also provide an impetus to act agentically. We found that the nurses’ COVID-19 trajectories were characterised by: getting by, getting out (job-hopping) getting needs met and getting organised. We call for more considered systemic support to be generated and consistently provided to nurses to ensure retention of nurses and the security of society to avoid exacerbating existing workforce shortages.

## Introduction

Pre-pandemic, pressure in the UK health services was high with nurses and midwives experiencing environmental conditions detrimental to their wellbeing [1, 2]. Researchers have highlighted that the reductionist, efficiency-based approaches to the National Health Service (NHS) workforce crises and a narrow focus on care delivery as a series of tasks to be completed, rather than the delivery of person-centred care, underestimates the workload involved in complex and high-risk nursing work [3]. The NHS has experienced structural issues in the form of prolonged exposure to underfunding and staffing shortages [3, 4]. Job satisfaction for nurses has been reduced through nursing shortages creating the adoption of task-based approaches to nursing, resulting in nurses compromising their ideals in regard to patient care which can lead to burnout and nurses leaving the profession [4, 5]. Researchers have highlighted the increased risk of poor mental health including burnout amongst nurses [1, 6]. The Office of National Statistics (ONS) data report the suicide rate among nurses, before the additional pressures of COVID-19, as 23% higher than the national average, with more than 300 nurses in England and Wales taking their own lives between 2011 and 2017 [1, 7].

In this context, the COVID-19 pandemic created a unique set of demands, which exacerbated these existing challenging working conditions for an already stretched workforce. Researchers have identified the extra pressures of working during the pandemic, including additional staff shortages, insufficient personal protective equipment (PPE), navigating unfamiliar clinical settings or new systems of care due to redeployment and lack of organisational support [8, 9]. Ustun [10] highlighted the dire effects of pandemic working conditions with primary traumatic stress, secondary traumatic stress, job burnout, compassion fatigue and moral injury as the main impacts.

In response to the onset of the pandemic in the UK, the Impact of COVID-19 on Nursing and Midwifery Workforce (ICON) study was established in March 2020. It initially involved the delivery of national surveys at three time points between April and August 2020, aiming to identify the psychological impact of COVID-19 on nurses [8]. Results from the survey indicated that during the first wave of the pandemic 45% of 2040 respondents exhibited symptoms indicative of post-traumatic stress disorder (PTSD) and personal and workplace factors were associated with adverse psychological effects [8]. This initial ‘parent study’ led to the development of an in-depth longitudinal qualitative study, from which our interviewees were sampled this paper reports, to better understand nurses lived experiences of working at the front-line during the pandemic and their pandemic trajectories.

### Transition and liminality literature

Longitudinal qualitative research provides researchers with the opportunity to study fluctuations and changes in participants’ accounts, which takes into consideration individuals’ ongoing, processual sense-making about their lived experiences, fluid identity constructions and life transitions [11]. The unique working conditions and pressures brought about by the context of the COVID-19 pandemic can be considered an occupational disruption to which the rapid workplace changes created an uncertain transitional or liminal period in the working lives of nurses in the UK [12, 13]. Liminality is frequently viewed to be a state that denotes a position of ambiguity and uncertainty as an individual is betwixt and between [14]. In a liminal state a person may be between two identity constructions as they are neither one thing nor the other [15]. A transition can be considered a change from one way of being or situation to another and can be gradual, or in the case of COVID-19, sudden and abrupt. Pertinently, Kralik et al. [16] define transitions as the process of convoluted passage during which people redefine their sense of self and redevelop self-agency in response to disruptive life events. At the time of writing, nurses in the UK continue to nurse COVID-19 patients with nurse retention highly problematic and staff shortages growing leading to national strikes. Therefore, it is unclear whether the change to working conditions wrought by the pandemic is temporary or permanent, and if the latter, what these working conditions will transition to, and what this will mean for nurses in the future.

Such transitions, or organisational disruptions, generate change in individual’s attitudes and beliefs as well as to organisational processes [17]. In this paper we are interested in notions of change through time, which illuminates the process of change whilst detailing the complexities of the journey [18]. Corden and Millar [19] highlight how consideration of change through time provides insight into how people organise their daily lives and think about the past and future. As Thomson et al. [20: 337] note, longitudinal studies of transitions can recognise that transitions are differentially experienced and interpreted and can seek to gain insight into ‘the subjective experience of personal change’.

Longitudinal studies of transitional times have employed narrative and biographical approaches to explore processes of social change with many endeavouring to capture the complexity of individual lives and the relative impact of events within them [21]. The combination of structural conditions brought about by the COVID-19 pandemic and nurses’ individual responses to the organisational disruptions are explored in this paper.

### Critical moments and biographical disruption

Pertinent to this paper are ideas of ‘fatefulness’ [22] and biographical ‘choice’. With his conceptualisations of ‘fateful moments’, Giddens [22: 243] attempted to pinpoint the moments ‘at which consequential decisions have to be taken’ which he argued create an empowering experience, with implications for self-identity, not just for future situations. Using this notion, Thomson et al. [20: 337] examined ‘critical moments’ in young people’s lives and reported that many of the young people experienced the same ‘transition’ points but their individual responses to these were very different. Thomson et al., [20: 351] attributed this difference to not all young people having the ‘requisite resources and opportunities’, even though the majority did speak the language of choice, control and agency over their lives.

Parallels can be drawn between these notions, which demarcate individuals’ biographical trajectories, and those that appear in medical sociological literature, for example Bury’s [23] use of the term biographical disruption. Conceptualised as a state in which a patient is put into after an official diagnosis, biographical disruption aims to understand individuals’ biographical routes through which they try to interpret themselves and their lives with a chronic condition and the experience of living with a chronic disease [23, 24]. For Bury [23], a biographical disruption separates life in two: before and after a diagnosis of a serious and or chronic illness or other major events such as death of a loved one or divorce. Other researchers have argued that biographical disruptions are more in line with Giddens [22] and Thompson et al.’s [20] conceptualisations of a fateful moment, as a diagnosis of a chronic illness can be analysed in the context of a lifetime [24]. Therefore, biographical disruptions can be perceived as changing ‘the course of life and every obvious personal element’ and is ‘subjectively experienced’ [24: 800]. For nurses, COVID-19 can be viewed as a biographical disruption, as the pandemic imposed rapid change, not only their personal lives, but also in their working lives. The uncertainty of the trajectory of the pandemic forced nurses into a liminal state in the covid period between pre and post-COVID-19. This liminality continues.

### Coping and adapting

Through data analysis, we identified resonance with literatures examining the trajectories of those living in poverty and we draw upon this [e.g. 25–27]. This literature examines the lived experience of individuals, predominantly women, through accentuating structural circumstances. Whilst unquestionably different, we argue that parallels exist between ‘existing’ under the harsh structural conditions of poverty with associated psychosocial effects and the harsh structural conditions endured by nurses working at the frontline in the NHS during the COVID-19 pandemic and the psychosocial consequences of these. Poverty is an economic adversity involving psychological hardship. Similarly, for nurses working during the COVID-19 pandemic, organisational difficulties existed as did psychological hardship. We are specifically interested in how Lister’s [26] poverty theory recognises an individuals’ capacity to act within the constraints of environmental circumstances. In adapting and applying her theory to the organisational context of healthcare services in the UK, we explore the capacity of individual nurses to act agentically within the structural context of the organisation and wider political health sphere in which the organisation is framed.

Lister’s [25, 26] four types of agency are useful to delineate nurses agency during the COVID-19 pandemic. The four forms of agency include: ‘getting by’ (coping and managing), ‘getting back at’ (everyday resistance), ‘getting organised’ (political responses), and ‘getting out’ (trajectories of change)’. Categories which involve movement, such as ‘getting out’ are transitions, but it is important to recognise that not all experience over time is about change in situation or circumstances. Experience over time also ‘involves living with what is, responding to relatively minor changes, or even avoiding change’ [26: 535]. The processes involved in getting by over time can be considered more effectively in qualitative than in quantitative data [26], and drawing on this work, we have used our data to explore how, and in what ways, nurses managed and adapted over the trajectory of the pandemic.

We have explored some key concepts from the sociology of transitions, poverty and medical sociology literature that have been developed through the use of mostly longitudinal qualitative research involving the study of dynamics and consistency of individuals’ biographical trajectories. These include transitions as movements from one state to another, coping with and adapting to situations or states over time, and trajectories of cumulative patterns over time. We now consider these concepts in relation to the data gathered from qualitative longitudinal data generated with nurses in the UK during the COVID-19 pandemic. The longitudinal interview study reported here examined in-depth nurses’ experiences of working at the frontline of COVID-19 and the impacts of the pandemic on their psycho-social wellbeing since March 2020. The aim of this paper is to explore the dynamics, transitions, adaptions and trajectories of nursing at the front-line in the UK during the COVID-19 pandemic using a social constructionist approach viewing realities as being constructed in a relational context and with multiple insights.

## Methods

**Fig 1:**
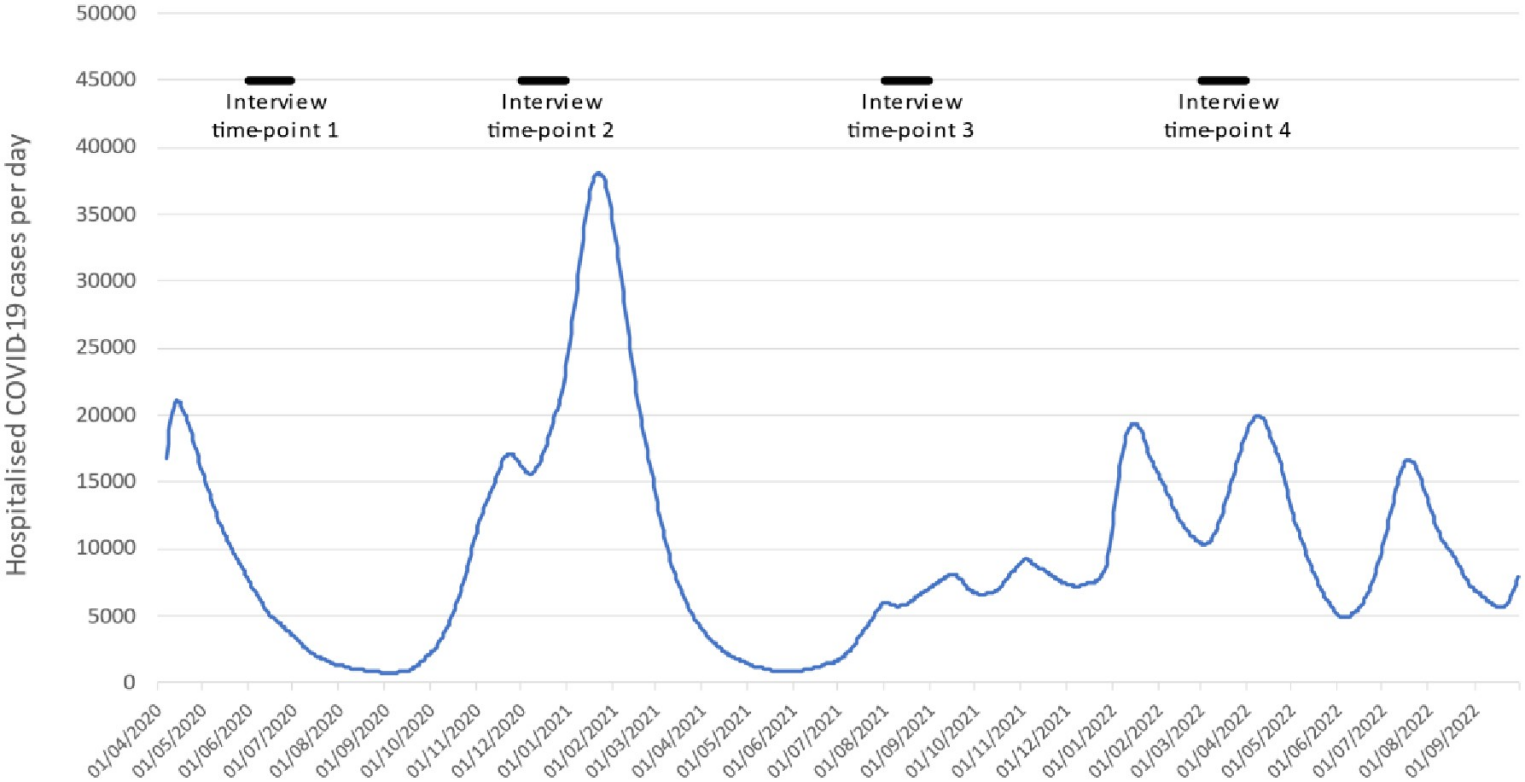
Interview time-points in context of UK hospitalised COVID-19 patients.

Narrative interviews were undertaken with 50 nurses, divided into two samples, with the aim of understanding their experiences during in-depth interviews over the trajectory of the pandemic. Sample 1 (n = 27) took part in 4 interviews, over 20 months, the first in July 2020 (n = 27). Twenty-five were interviewed in December 2020, 26 in August 2021 and 21 were interviewed for the final time in March 2022. Due to the low numbers of non-white, student nurses, care home nurses and community nurses in the first sample we decided to extend our participant population to include larger numbers of these nurses. Interviews with Sample 2 (n = 23) began after the second wave of COVID-19 in the UK in August 2021 and 19 of the nurses in this sample were reinterviewed in March 2022. The interviews were spaced fairly evenly over the course of 19 months and coincided with several peaks in the numbers of hospitalised cases [28]. Fig 1 shows our interview distribution time points in the context of the number of hospitalised patients across the UK, although it is important to be mindful that regional variations occurred in regard to the dates of the peaks and subsequent recovery trajectory.

Participants were recruited through an opt-in method with participants who had completed the parent study national nurse and midwife surveys and expressed an interest in being contacted to take part in qualitative interviews about their COVID-19 experiences [8]. All participants were emailed with a participant information sheet and consent form and given 14 days to respond. Purposive sampling occurred with both samples. The addition of sample 2 ensured a breadth of participants’ experiences with nurses working in a range of settings and specialities, differing experiences and levels of seniority. Nurses were based in varying geographical locations throughout England, Scotland, Wales and Northern Ireland and were also sampled from varying ethnic groups and age ranges. Out of the 50 participants, 2 were men. More information about the demographics of the participants is provided in Table 1 below. Ethical approval was received from the (Anonymised University). Interviews were conducted by all authors, each researcher interviewed the same participant at subsequent interviews. All the interviewers were female except one. All those who undertook interviews are experienced in conducting and analysing qualitative research and also have expertise in conducting interviews on distressing topics (please see 30 for more information on the interviewing process). A list of wellbeing resources were made available for participants after the interviews and researchers were able to ‘check in’ on participants.

**Table 1:** Participants characteristics with trajectories.

Participant name	Ethnicity	Age	Grade at start of COVID-19	Redeployed or student during COVID-19 wave 1	Clinical setting	Interview time-point 1	Interview time-point 2	Interview time-point 3	Interview time-point 4
Chloe	White British	51–55	5	.-	Care home	x	x	1	1
Amanda	White other	51–55	5	-	Care home	x	x	1	x
Jemima	White British	61–65	2 to 4	-	Care home	x	x	1	2
Raheem	Black African British	56–60	7	-	Care home	x	x	1	3
Amie	Mixed White and Asian	56–60	8b	-	Care home	1	4	1	x
									
Lucy	White British	51–55	6	-	Community	x	x	1	x
Fiona	White British	46–50	6	-	Community	x	x	1	1
Maddie	White British	36–41	2 to 4	Student	Community	x	x	1	1
Sophie	White British	46–50	6	Redeployed	Community	1	1	1	2
Edie	White British	61–65	5	-	Community	x	x	4	x
Tilly	White British	41–45	8c	-	Community	x	x	2	1
Gaby	White British	56–60	6	Redeployed	Community	3	1	1	2
Tanu	Black Caribbean	56–60	6	-	Community	x	x	1	2
Sue	White British	46–50	8a	-	Community	1	2	1	1
Jess	White British	61–65	2 to 4	-	Community	x	x	2	1
Lizzy	Mixed White and Asian	56–60	5	-	Community	3	x	x	x
Lily	White British	51–55	5	-	Community	x	x	1	3
Mia	White British	56–60	6	-	Community	1	4	1	1
									
Ellie	White British	31–35	7	Redeployed	Hospital	1	1	1	x
Caitlin	White British	41–46	2 to 4	Student	Hospital	x	x	1	1
Jo	White British	31–35	8a	Redeployed	Hospital	1	1	1	1
Sherie	White British	51–55	6	Redeployed	Hospital	1	1	1	1
Mary	White British	51–55	7	Redeployed	Hospital	1	1	1	1
Sandra	White British	41–45	7	Redeployed	Hospital	1	1	1	1
Saffron	White British	51–55	8c	-	Hospital	1	1	1	1
Lara	White British	56–60	5	-	Hospital	1	1	1	1
Eve	White British	36–40	2 to 4	Student	Hospital	x	x	1	1
Laura	White British	31–35	6	Redeployed	Hospital	1	2	2	2
Camila	White British	46–50	7	Redeployed	Hospital	1	1	3	2
Isla	Mixed ethnicities	36–40	6	-	Hospital	1	x	2	2
Tessa	White Irish	46–50	7	Redeployed	Hospital	3	1	2	x
Isabella	White British	51–55	7	Redeployed	Hospital	1	1	1	2
Annabel	Black African British	26–30	5	-	Hospital	x	x	2	1
Rachel	White British	31–35	7	Redeployed	Hospital	1	1	2	1
Amelia	White Other	36–40	6	Redeployed	Hospital	x	x	1	2
Zoe	White British	51–55	6	-	Hospital	x	x	1	2
Sarah	White British	26–30	5	Redeployed	Hospital	1	1	1	3
Amber	White British	56–60	5	-	Hospital	x	x	1	3
Ria	Black African British	56–60	8b	-	Hospital	x	x	1	3
Bethan	White British	31–35	7	-	Hospital	x	x	1	3
Peter	White British	20–25	6	Redeployed	Hospital	4	2	2	2
Sephy	White Other	20–25	2 to 4	Student	Hospital	x	x	4	x
									
Catherine	White British	46–50	9	Redeployed	Mental health	1	1	1	1
Alison	White British	46–50	8a	Redeployed	Mental health	1	1	1	1
Julia	White British	56–60	8a	Redeployed	Mental health	1	1	1	2
									
Helen	White British	46–50	7	-	Midwife	1	1	1	x
Elizabeth	White British	41–46	7	Redeployed	Midwife	x	x	1	1
									
Louise	White British	46–50	8b	Redeployed	Research	1	1	1	1
Kiya	Asian British	36–40	7	Redeployed	Research	x	x	1	1
									
Becky	White British	46–50	9	-	Social Care	1	1	1	1

In the UK nurses have standardised grading with band 5 the entry level for registered nurses and band 6 for many registered midwives. Nurses who work at higher grades have achieved a higher level of seniority and greater pay.

Key

1. Getting by

2. Getting out

3. Getting needs met

4. Getting organised

X No interview

The interviews were conducted online, mostly at the participants’ home, with only the researcher and participant present during the interviews. During the interviews a non-directive interview topic guide was used loosely with the researchers inviting participants to ‘tell me what happened’ and allowing participants to speak uninterrupted until their story ended. Therefore, we used the interview guide as prompts only where needed, instead eliciting narratives and following respondents ordering and phrasing to allow participants to discuss areas they perceived to be relevant [29]. Longitudinal qualitative methods, in the form of repeat interviews, were used to identify and characterise personal trajectories as the pandemic progressed.

After each interview participants, along with the list of wellbeing resources, participants were given the opportunity to speak with a member of the research team. The research team supported each other with the emotional effects of interviewing which were at times demanding and distressing [30]. Interviews lasted from 45 to 90 minutes with one participant undertaking a further 90-minute interview after the first interview to provide more details and clarification. After verbatim transcription, all transcripts were held securely on password-protected computers. Anonymity was ensured through the removal of identifying information and allocation of pseudonyms.

As with all longitudinal research this study experienced an attrition rate, although this was low, with 5 nurses only interviewed once. 4 of these nurses were from sample 2 and their attrition from the study can perhaps be attributed to the timings of the interviews for this sample, with their participation in the research occurring during a context of lower COVID-19 hospital admissions. It is important to be mindful that longitudinal data was not collected for five participants. These participants’ details are included in the results table below, but their narratives do not feature in this paper.

### Data analysis

Interviews were inductively analysed for themes and a narrative analysis was then conducted to preserve the form of each participant’s narrative [29]. Therefore, we firstly used NVivo 12 to organise data and develop inductive codes and themes across the datasets and this production and application of emergent codes enabled the reflection of the view of participants in, what can be conceptualised to be, a traditional qualitative manner. Pen portraits, or interview summaries, were also produced which helped to avoid fragmentation of the data [31]. As per Riessman’s [31] description of narrative analysis, we strove to identify segments of text that take the form of narrative, and examined structural and linguistic features to analyse how they support particular interpretations of the lived experience of each research participant [31]. Therefore, we identified whole stories instead of segments of text and we used Muller’s [32] five overlapping stages of narrative analysis to guide the development of the pen portraits. The production of secondary level themes, which were used along with the pen portraits, aided our longitudinal holistic approach to analyse each participant [29]. The lead author examined each participant’s interviews and then compared with data from other participants at the same time point. Comparisons then took place across all time points [33]. Two further authors carried out the same analysis on a sample of transcripts [9].

We have completed the Consolidated Criteria for Reporting Qualitative Research (COREQ) checklist for comprehensive and explicit reporting [34]. Important aspects of the study have been reported (research team, methods, context, findings, analysis and interpretation) with an audit trail to allow for critical evaluations of the study’s trustworthiness.

## Results

**Fig 2:**
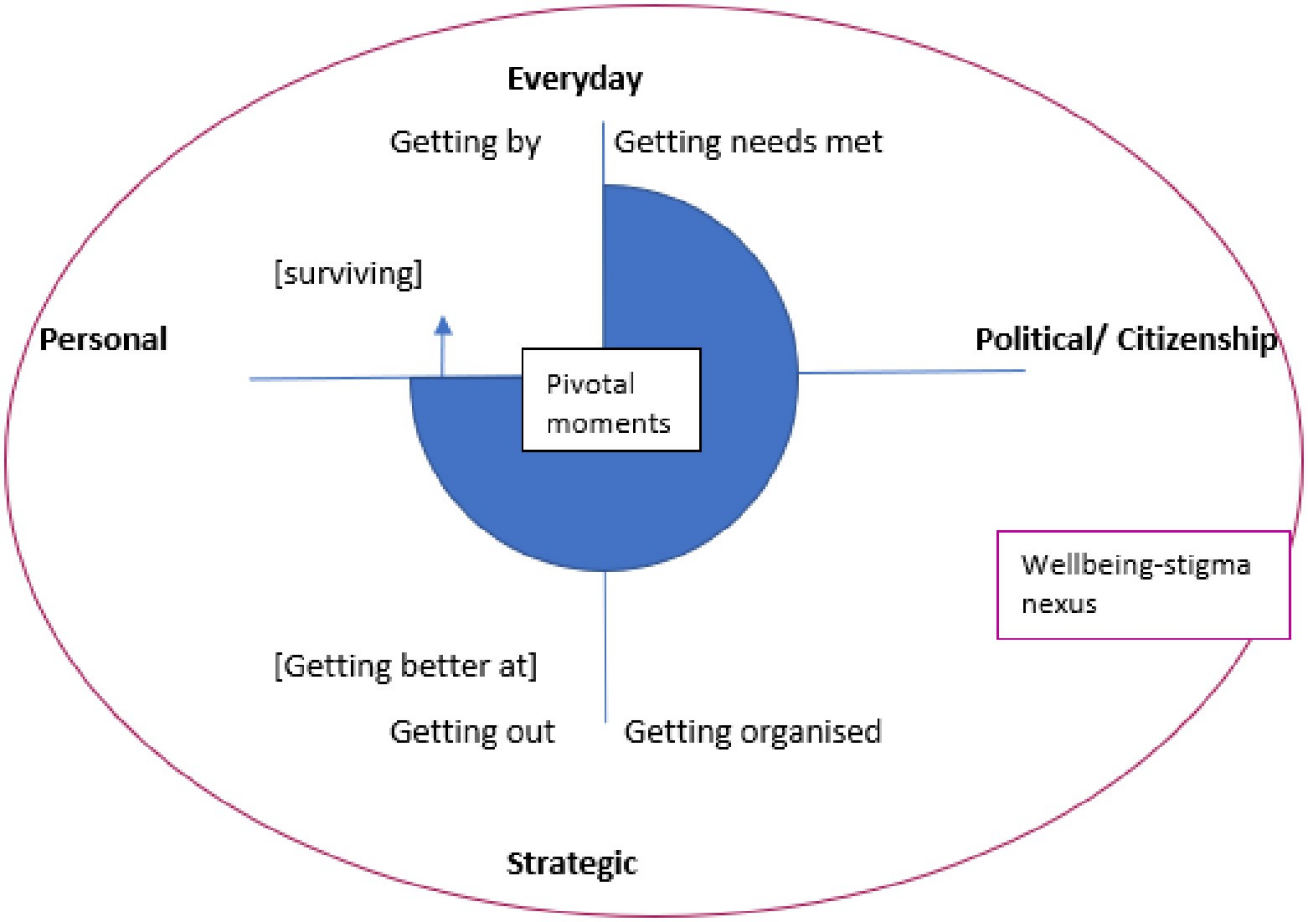
Forms of agency.

The organisational disruption caused by the pandemic impacted greatly on the nurses in our study. Collecting data longitudinally enabled us to map the changes and similarities of the interviewees’ trajectories over the pandemic. These were mapped using an adapted version of Lister’s poverty trajectory criteria (getting by, getting needs met, getting out and getting organised), to understand whether there was a dominant trajectory or a pivotal moment that stimulated action by the nurses (Fig 1). The typology of agency depicted in Fig 2 is based on two continua: from the more ‘everyday’ to the more ‘strategic’, which can be seen to reflect the strategic significance for people’s lives of the choices they make (vertical axis) and from the more personal to the more political (horizontal axis) [25, 26]. The taxonomy categorises actions not actors, so that any one individual could be exercising multiple forms of agency. We have adapted Fig 2 from Lister [26]. Our additions include ‘getting needs met’, ‘pivotal moments’ and the ‘psychological vulnerability-stigma nexus’ in which the forms of agency are situated. Table 1 combines nurses’ demographic characteristics with their pandemic trajectories. For the purposes of this paper, we categorised the nurses’ trajectories by the category most dominant in their pandemic-trajectory narrative. The trajectory mapping shows that the majority of the nurses remained as ‘getting by’, which although this may appear to be a passive position, the nurses still utilised their agency to employ strategies to be able to cope with the challenges involved in getting by. A further group of nurses demonstrated their agency through changing their working environment (getting out or job hopping). The mapping also reveals that there was a small a group of nurses whose trajectory was characterised by ‘getting their needs met’ which they did by prioritising their own wellbeing through sick leave or unpaid leave. Other nurses used their agency to politically resist and question the organisational changes. The nurses in these later groups group had pivotal moments, similar to Giddens’ critical moments [22], within their trajectories that forced them to mobilise from a seemingly passive position (getting by) (although the nurses did actively use their agency to manage ‘getting by’), to one of visible action which often involved taking sick leave or moving job. Plotting our data into Table 1 allowed the exploration of global patterns within our data set. We now analyse the nuances and fluctuations within the trajectories with our adapted version of Lister’s [26] criteria.

### Temporal contextualisation of our findings

Fig 1 plotted the interview time-points in the context of the very high rates of UK COVID-19 hospitalisations which occurred just prior to the first, and just after the second, wave of interviews. The figure shows that hospitalisations were much lower in the third and fourth wave of interviews conducted in 2021/22. As indicated in Table 1 which details participants’ pandemic trajectories, very few participants do anything other than ‘getting by’ in the 2020 interviews. It can be argued that, as we have explored in Maben et al. [9], at the beginning, the shock of being in a pandemic situation, along with the heavily altered workload which featured ‘deathscapes’, where many patients died due to the limited patient treatment and intervention options nurses, strategic as well as everyday agency was undermined. The majority of the nurses referred to having extremely depleted personal resources at this time, were exhausted by the very struggle to ‘get by’ and overwhelmed by feelings of hopelessness, powerlessness and lack of control. However, as the pandemic progressed during 2021/2022, the prolonged strain of ‘getting by’ and the sheer grind of daily work in COVID-19 conditions meant nurses became increasingly desperate and strived to make changes to their circumstances. It is clear that ‘getting needs met’, which includes taking sick leave, was more common in the March 2022 interview accounts, with many nurses who had time off during this period doing so for mental health reasons.

When examining how nurses’ work settings, redeployment during the first wave of COVID-19 and grades (or the pay scale they are on) map onto their pandemic trajectory characterisations there appear to be some patterns in the data. In this context being ‘redeployed’ means being assigned to perform a role in a new setting or area, (usually COVID-19 wards or intensive care units—ICU), roles which are different to the one the nurses were usually employed to work in. Although many of the nurses who were redeployed continued to ‘get by’ high numbers also ‘got out’ whilst some got their needs met. Elsewhere we have explored how traumatic redeployment, which often involved non-ICU nurses being sent to work in ICU with minimal training was for some of our participants (9). Being unaccustomed to working with so many critically ill patients, where death occurred frequently could mean that nurses’ defence mechanisms were less developed and therefore the work was more traumatising. The grade the nurses were on (effectively their level of seniority within their organisation) appeared to have some impact, with more high grade nurses able to ‘get on’ than those who were in grade 5–6.

Community, hospital and care home settings appeared to have similar rates of ‘getting out’, ‘getting needs met’ and ‘getting organised’ by the fourth interview time-point. This is the case even though no care home workers were redeployed and very few community workers were. It could be argued that community and care home work was similar in nature to the work experienced by those nurses who were redeployed, with common characteristics including working with patients with high mortality rates with fears about their own safety due to lack of PPE. Although with smaller proportions of participants, it would appear that the most protective work settings for nurses during COVID-19 were mental health, midwifery, research and social care. Some of the nurses working in these areas were redeployed but were able to return swiftly to their previous roles which had experienced little change. We will now go on to explore in depth what each category meant for the nurses who experienced them.

### ‘Getting by’

For the majority of the nurses, their trajectories during the COVID-19 pandemic were marked by ‘getting by’. Research (e.g. 9), has recounted how difficult and distressing nurses work during the COVID-19 became. For example, Ellie, who was redeployed during the first and second waves of the COVID-19 pandemic described how she felt she just ‘mucked in’ and ‘cracked on’ to cope with the unrelenting nature of working in the NHS during the pandemic:

ICU during COVID it was really quite harrowing. You know, I’ve got a lot of ICU experience and there was some days when I felt completely overwhelmed with what we were seeing [but] (…) in the thick of it you just sort of feel like you’re in the trenches, you’ve all got to muck in, you just crack on.

**Ellie**, **interview time-point 1**

For Ellie, similar to many of the interviewees, working at the front line during the pandemic during her redeployments, was characterised as being in a war (‘in the trenches’) and her working environment was something she had minimal choice over. Ellie discussed employing coping strategies, such as not sharing her experiences with her friends and family to be able to keep going. Previous research has highlighted techniques individuals employ to enable to be ‘seen to be coping’ [35: 121] including keeping up appearances and withdrawing from social interactions to avoid being shamed [26]. This task was made easier during the imposition of COVID-19 restrictions in the UK.

Lister [26] linked ‘getting by’ to the avoidance of stigma or shame arguing that the ‘very fact of getting by is sometimes used as evidence of not being [the label]’ [26: 149]. For nurses, not being able to cope with poor or worsening wellbeing has frequently been highlighted as a source of stigma [1]. Similar to other studies of nurses and doctors in the NHS e.g. [36] we found that many nurses described a workplace culture of needing to appear invulnerable, where work-related stress and distress were normalised and taking time off for physical or mental health is poorly tolerated:

I work with a nurse who struggled with her mental health before COVID. She had postnatal depression and then going in to COVID that, kind of, ramped up (…) from all the team there was a lot of support for her, but from management and line managers (…) every time she was struggling they put it down to her mental health. She couldn’t have a cold without it being blamed on her mental health, and she was really, she felt very much segregated by the management because of that.

**Caitlin**, **interview time-point 4**

The ‘segregation’ of the nurse, which was compounded during the pandemic, is one of many stigmatising actions, related to guilt and fear of employment-related discrimination, negative social judgement, or being admonished or shamed. The ‘psychological vulnerability-stigma nexus’, which some of our nurses referred to as increasing during COVID-19, may have delayed seeking help for some and often led to many participants working when they were unwell (presenteeism). Nurses referred to the pressure ‘to be there’ ‘for their colleagues’. Similar to Lister’s [26] ‘poverty-shame nexus’ we believe a ‘psychological vulnerability-stigma nexus’ exists within nursing, and it was certainly prevalent in our data. Driven by a process of ‘othering’ where those who can ‘get by’ or cope treat those who cannot ‘get by’ as different, it is a process of ‘differentiation and demarcation that draws a line between ‘us’ and ‘them’, which establishes, maintains and justifies social distance’ [26: 142]. This process can be reinforced through stereotyping and stigmatisation. ‘Getting by’ or ‘carrying on’ meant the nurses were engaged in the daily task of performing resilience and endurance. Resilience during COVID-19 has been problematised elsewhere [37]. Due to the ‘psychological vulnerability-stigma nexus’, for many nurses ‘getting by’ became a task they had to do. Our data showed that for many nurses who were engaged in the task of ‘getting by’ wellbeing resources which were often provided by their employers were not used. Due to the ‘psychological vulnerability-stigma nexus’ which created a culture of invulnerability, when nurses felt they really needed to access wellbeing resources such as counsellors, they frequently accessed these resources from locations outside of their employers, such as charities or unions, confidentially. The agentic strategies employed by nurses to ‘get by’ were often social and involved talking to their colleagues who had ‘been through the same thing’, either through face-to-face meetings when allowed with social distancing rules or via social media groups [9]. On a more individual level nurses also frequently talked about keeping mementos of their COVID-19 work, for example one nurse referred to keeping a COVID-19 box that contained items such as a memorial service for a colleague who died after contracting COVID-19. Another nurse, Sherie, referred to doing something similar:

I’ve still got on my desk the printouts of all the patients I did video calls with [during the first wave of COVID-19] (…) And lots of them died and I cannot bring myself to throw the paper away (…) which is probably not really healthy actually, is it, but, yeah, it feels important to remember them (…) I just think of all their names. I can still reel off names of people who died and it’s, like, it was difficult, but not as difficult for the people who lost people.

**Sherie**, **interview time-point 4**

Sherie worked as a family liaison nurse during the pandemic and was responsible for calling relatives who were not allowed (due to government imposed pandemic laws) to visit even dying patients during the first wave of the pandemic. Sherie attached great importance to remembering not only the patients who died, but also the plight of their relatives who had to endure the remote deaths of their loved ones being able to visit them. She questioned whether keeping her lists of patients was good for her wellbeing (‘it is probably not healthy’) but her view that it was ‘nice’ to keep them underlies the importance to her that what she, and others have been through should not be underestimated or forgotten.

As the pandemic wore on the daily grind involved in ‘getting by’ became more obvious to some of the nurses.

I was very aware [of the pressures of nursing] going into it, but I just don’t see how it’s sustainable the way it’s going. I don’t see how the NHS can carry on, which breaks my heart because I’m a big supporter of the NHS (…) the patients/the service users are so demanding and rightly so, but we can’t keep up. And it’s demoralising to not be able to give the care that you want to (…) it does make me question it because also that impacts on my family.

**Caitlin**, **interview time-point 4**

Even though many of the nurses were ‘getting by’ they felt unsupported, particularly in the wake of the disruption brought by over two years of the NHS trying to cope with the extra pressures of COVID-19 and staff shortages. We argue that remaining in post was an active choice for the nurses. When nurses had a realisation that they were undervalued they would feel prompted to take action. In the next section, we explore how some nurses decided to change, to stop ‘getting by’ and remove themselves from the stress of the situation/environment.

### Getting out (or job-hopping)

The interplay between agency and structure in shaping individual trajectories, facilitated individuals to ‘get out’ either by leaving nursing or by ‘job-hopping’ [38–40]. Previous literature has identified frequent moves by newly qualified nurse graduates as ‘job-hopping’, a term used originally by Kramer [38] to describe nurses who constantly look for the ideal environment, one that aligns with and practices the nurses’ own values. Nurses frequently used their agency to undertake job-hopping after experiencing a ‘fateful’ or ‘critical’ moment where they were able to evaluate their working lives. For example, Isabella reported experiencing, what she called, a ‘pivotal moment’:

The new manager (…) we had a meeting with the ward’s management (…) it just felt like a bit of an ambush, and there was just no recognition of all that we do, and I work, I slog from the minute I arrive to the minute I leave (…) I just felt it was very much, like, “Yeah, but you’re not doing enough.” (…) and I thought, ‘I’ve got nothing else to give’ (…) I just felt overwhelmed and undervalued. I went home that evening, my son was home from Uni and I just cried in his arms.

**Isabella**, **interview time-point 4**

Isabella’s realisation that whatever she did would never be enough, led her to consider what had previously been unimageable in her long career as a nurse, leaving the NHS. Although Isabella did not have a supportive manager at this time, a hospital consultant recommended she attend a job interview at the private hospital where he also worked. She took the job when it was offered to her, describing her motivation as being to seek recognition and be valued for her work:

I thought, “No one else has seen the value in me. I’m going to see it in myself. I’m going to go for this.” I went for it, and I’ve got the job and I left the NHS after 35 years. You know, it was in my bone marrow. I just didn’t think it was coming, but I started the job two weeks ago and I’m loving it.

**Isabella**, **interview time-point 4**

The liminal nature of this period had enabled Isabella’s identity to shift from being an NHS worker (‘it was in my bone marrow’) to working in a privately owned hospital. She very firmly attributed her agentic decision to the ‘pivotal moment’ that lead her to deeply question the ethical implications of how her organisation treated her. In the nurses’ narratives, we found that when pivotal moments were mentioned, they tended to be used as justification for the change to their lives that the nurses made. For so many of the nurses, such as Isabella, their work was fundamental to their sense of self, and making the transition to another employer was disruptive and needed to be almost excused by the occurrence of a pivotal moment in order to be made justifiable. Giddens’ [22] notion of fatefulness is therefore useful in making sense of Isabella’s account, as it illuminates the centrality of self-identity in the progression of biography and allows the exploration of a process of identity revision. However, by privileging individual choice, this theoretical approach to understanding the significance of working through the pandemic obscures the wider organisational processes that may have constrained choice in practice.

‘Job-hopping’ was frequently described by nurses as an attempt to find psychological health protection and self-autonomy. For example, Camila described experiencing difficulties in her role after her redeployment. After contracting COVID-19 she suffered from long-COVID and took a period of sick leave. She described feeling undervalued in her hospital-based role and struggled to cope with her long-COVID symptoms in a pressurised environment. When offered a local, community based job Camila did not hesitate to take it:

So I’ve changed job (…) I got a phone call from my local practice/my local surgery who knew that I was keen to be a nurse practitioner and they said, “Do you want a job? (…) Come and have a chat,” I did and I was offered a job—a contract basically stuffed through my letter box in the next couple of days, and yeah, I took it. The money’s terrible, but I took it (…) Yeah, so I’m a nurse practitioner now, and I’m working 10 minutes’ walk from home.

**Camila**, **interview time-point 4**

Camila’s experience of feeling undervalued, a push factor, prompted her to consider a completely different role, a pull factor. Other nurses described ‘job-hopping’ to try to pursue roles that aligned more closely with their identity as nurses including their values and their visions of compassionate care delivery which some said was compromised in the pandemic [9]. For example, Sophie, who went to work in a different nursing sector related:

[Its been] amazing, plain sailing. It’s been fantastic (…) Anyway, so I like the job, I like the team, I like the work. But above all, it’s extremely well resourced (…) with senior staff. It’s absolutely amazing (…) And so I am supported to provide high quality nursing care to my patients. Unbelievable. I haven’t had that for ages.

**Sophie**, **interview time-point 4**

Sophie’s narrative indicates that by job-hopping’ she could stay in nursing and find a team that allowed her to nurse patients in a supportive manner and to give patients the care she believed they should have. This was tempting for many nurses in our sample. The ability to fulfil their nursing ideals in practice can be viewed as an intrinsic part of many nurses’ identity. Through the concept of ‘job-hopping’ Kramer [38] revealed that there is mobility within nursing, with some nursing roles short term. For most of the nurses in the current study, job-hopping was a successful practice, with the nurses feeling happier in their new roles. However, these roles had only been adopted a short time prior to their final interviews and it is hard to know if the nurses would grow to feel frustrated with their new roles and ‘job-hop’ again.

Some of our participants made explicit links between job-hopping as a coping / adaptation strategy, even a form of preventative psychological self-care, which enabled them to avoid taking sick leave. For example, Annabel, reported leaving ICU after the first wave of the pandemic to work as a community nurse. After working for some months in the community and then reapplying to become an ICU nurse again, Annabel was able to evaluate the process she had gone through:

What other people would have done is probably identified that they weren’t coping and that actually they need a break and spoken to the manager and said, “I think I’m going to have to go off sick because (…) I’m not functioning as a nurse and, you know, it’s not good for me,” but instead, [I] went and got another job (…) it’s that stigma of (…) I don’t want to seem like the one who can’t manage (…) because that is a weakness (…) but when you’re working in an environment like ICU where you need to be quite strong and pull it together and be emotionally resilient, to be seen as weak and not being able to cope is not what I’d want to be associated with me as, which, rightly or wrongly (…) we hold ourselves with this great big pressure that we’ve got to be okay

**Annabel**, **interview time-point 2**

The ‘psychological vulnerability-stigma nexus’ with the associated culture of invulnerability made some nurses practice ‘job-hopping’ as a strategy which enabled them to have a break from those stressful environments which may have contributed to their poor psycho-social wellbeing. Annabel referred to feeling unable to tell anyone about her poor psychological wellbeing which had declined as a result of working in ICU during the pandemic. Her coping mechanism of ‘job-hoping’ enabled her to successfully have a break from the setting that had so powerfully impacted her mental health (which included her suffering from symptoms of PTSD including flashbacks) without anyone knowing. She recognised that taking time off due to stress would be seen as a ‘weakness’ and her urgent need to keep that label as something that others experience, not something she would experience, lead her to ‘job-hop’ for some respite. After some months away Annabel began to feel that her identity was bound to ICU and she reapplied for her old job and got it back:

I just feel that I’m back to me pre-COVID, back in ICU, which is just lovely and it’s definitely where…., that’s the sort of nurse I am. So I was so grateful for the break, you know, changing roles (…) and I needed that, but that also made me see that that’s not the type of nursing that I enjoy (…) I’m feeling stronger, I feel like I’m back in the right place.

**Annabel**, **interview time-point 2**

Annabel’s return to ICU enabled her to fulfil her true identity—as an ICU nurse. Others nurses we interviewed who confided they were suffering with their mental health as a result of the pandemic also related their desire to ‘job-hop’ as a way of continuing to be a nurse whilst trying to shed the negative facets associated with their current roles. However, ‘job-hopping’, or the movement of individuals back and forth, and sometimes back again, perhaps illustrates that nurses might often be looking for something that does not exist. In the next sections we explore how some nurses chose to prioritise their own wellbeing during their COVID-trajectories.

### ‘Getting needs met’

In the context of a culture of invulnerability as described above, prioritising wellbeing through sick leave or unpaid leave by taking time for oneself can be viewed as a brave step. In this section we explore those nurses who felt able to use their agency to take a break during their pandemic-trajectory, whether that was through sick leave or unpaid leave. We are mindful that for some nurses who were very unwell during the pandemic, such as Ria, who had chemotherapy, sick leave was not an active choice, but was a necessity. In Lister’s [26] conceptualisation this category was called ‘getting back at the institution’ which involved a form of everyday resistance aimed at survival. Key to this resistance was a resentment against the system [26]. We argue that for many nurses a realisation of resentment of this kind frequently equated to a ‘critical’ or ‘pivotal’ moment. For the nurses in our study unpaid leave or sick leave was frequently reported as taken when the nurses actively decided to privilege their own psychological wellbeing above the system’s (their employers). Many of the nurses related feeling anger at how their employers had treated them and these feelings of being undervalued, disillusioned and resentful for many, seemed to play a key role in their decision to take a break. For example, during his first interview Raheem, who worked in a care home, referred to the profit driven, rather than patient centred, environment he was working in during the pandemic:

The support is not there, especially now that staffing level, every day you go you are actually fighting and battling and struggling. (…) It’s about profit, not people, (…) so we are thinking about cutting costs here, cutting costs there. We don’t care about the standard of the care we give and that makes it really [hard], this is not how we started.

**Raheem**, **interview time-point 3**

Raheem’s realisation that profits were being privileged above residents’ care was a slow and gradual process, but one that both accelerated and solidified during the pandemic. Although not a critical moment, in that there was one key identifiable moment where this realisation took place, the gradual transformation of believing resident-centred care was prioritised to believing profits or money efficiency came first was accentuated, like many nurses, by the working conditions Raheem experienced during the pandemic. In his last interview Raheem referred to taking two months unpaid leave, which actually involved his resignation from his role:

So after everything happened, to be honest, I actually have to take a time out. And I travelled, I left the UK for almost about two months (…) And I was just thinking that is the end, you know, because everything was just too much. But (…) after I got a really good rest and (…) de-stress myself, I realised that where else am I going to go? So I came back (…) I think for me now, it’s about changing myself, changing my focus

**Raheem**, **interview time-point 4**

Raheem presented his decision to have a break as a necessity—it was something he ‘had’ to do. However, Raheem made an agentic decision to take an unpaid break from work. It is striking that he had to hand in his notice to be able to get a break, as the care home where he worked had consistently cancelled planned leave since the beginning of the pandemic. Although Raheem considered leaving the UK for good, the ‘pull’ of regular employment as a nurse was too great and he returned to the care home he had previously been working in. He returned to his job believing that *he* needed to change the way *he* dealt with difficulties, not that the job needed to change to provide him with more support to make his role easier.

The support of line managers appeared to make a difference to the nurses’ trajectories. For example, Raheem, who worked in a care home, was able to use his agency to take a break, even though he referred to not having supportive managers. Conversely, Bethan seemed able to take a break from her role due to the support of her manager. Bethan who took time off work with stress, emphasised agency in her narrative, along with a ‘pivotal’ or ‘critical’ style moment:

My line manager was very supportive, and she suggested taking some time off and I was like, (…) “I want to carry on. I feel like that’s admitting defeat,” (…) then actually the thing that changed it for me was reading a news website article written by a nurse who had experienced similar symptoms (…) just sort of seeing myself mirrored there and then seeing how kind of bad it got for her and I just kind of thought, actually, I want to do something about this (…) so, it was actually a decision I had to make (..) it took a few weeks really for me to go, “Okay, I can prioritise myself here.” Of course, you feel so guilty about taking time off when the unit’s so busy and your colleagues are all struggling so that was kind of tough (…) So I called the GP and explained I said that I’d been seeing the clinical psychologist at work and I had symptoms of PTSD

**Bethan**, **interview time-point 4**

Bethan’s ‘pivotal’ moment came when she read the online account and decided to act. Her desire to ‘do something about this’ to avoid being in the same position as the other nurse can be viewed as a brave step in a culture of invulnerability. She referred to the guilt she experienced about taking time off, but she characterised the ‘hard’ ‘decision’ she made to ‘prioritise’ her wellbeing as worthwhile. It is interesting that Bethan presented her decision to take time off (which lasted for at least 4 months) as a choice to prioritise her psychological wellbeing.

For many of the nurses who experienced a pivotal moment and felt able to prioritise themselves, the presence of a supportive manager who enabled them to exert their agency in this way was key. It is clear from Bethan’s narrative above that she felt supported by her manager who told her to take ‘some time off’. Other nurses also reported feeling supported by their managers, for example Lily who was not sure whether she would return:

They’re desperate to have me back but, you know, they don’t want me back, if you see what I mean, until I’m well (…) I think everyone’s really supportive, and I can’t see me going back to work for another month, if at all (…) it’s day by day, to be honest.

**Lily**, **interview time-point 4**

Lily took time off after having had further ill health concerns. However, in her longer narrative she related feeling undervalued as she fought to ‘get by’ at work with ill health. Therefore, after feeling unwell with her new health concerns, which included long-COVID, her resentment which had built up during her previous ill health episode led her to privilege her own health above that of her organisation’s wellbeing. This provided the impetus for her to use her agency and overcome the psychological vulnerability-stigma associated with taking time off. By refusing to be stigmatised, the nurses who took time off challenged the ‘othering’ they may have been subjected to through the ‘psychological vulnerability-stigma nexus’. We will now turn to notions of politisation during the pandemic.

### Getting organised

During the pandemic, particularly in the first wave in the UK, prevalent discourses called for solidarity and unity amongst nurses as they fought the enemy of the virus [41]. The prevalent hero discourses the nurses worked within during the pandemic placed intense pressure on them to work together ‘for the good of the NHS’ and not to enter into political or collective action [41]. Our data revealed that individual action was often fraught with obstacles and permeated by notions of ostracisation and stigmatisation that can occur to ‘whistle-blowers’. In this manner raising concerns or ‘whistle-blowing’ can be viewed to be closely linked to the ‘psychological vulnerability-stigma nexus’, as individuals are blamed for experiencing problems. During the pandemic when nurses felt able to speak out they frequently found that their organisations were not able (or did not want to) listen to them:

In the NHS you get told off for talking to certain people about certain things and raising certain issues in certain ways. And that’s just an absolute nightmare. You know, I was raising this issue about the fact that people weren’t getting told their [Covid-19] results over the phone, and it was almost being suggested that I was being insubordinate by raising that as an issue to my managers

**Peter**, **redeployed**, **interview time-point 1**

[I’d felt] absolutely awful because I’d had COVID, I’ve not been paid, I’d come back, working in all the PPE and the circumstances that we were in (…) So we we’re told you’re not allowed to go between floors, but then if they needed a nurse on another floor, oh well, it’s okay (…) But if you voiced your concerns (…) there was always a reason why it was happening, you know?

**Jemima**, **interview time-point 4**

The above quotes illustrate how the ‘psychological vulnerability-stigma nexus’ extended to a culture of not-raising concerns and not whistle-blowing. Peter and Jemima refer to being ignored when they raised concerns. This nexus meant the culture of invulnerability the nurses worked within was reliant on nurses staying quiet and not highlighting problems within the organisation. The agentic act of raising concerns can be seen to have acted as a pivotal moment for both Peter and Jemima as Peter soon left the NHS to take up a nursing associated role and Jemima ‘job hopped’ out of the private sector to work as bank staff in a hospital ward. Again, the nurses felt able to use their own agency to effect change, but the environmental conditions of the organisation were not optimal for these concerns to be acted upon, which caused feelings of disillusion resulting in the taking of an active decision to leave their organisations. We have explored the non-optimal environmental conditions nurses experienced regarding speaking out within their organisations during the pandemic in Abrams et al., [42]. It can be argued that raising concerns without these being acted upon by managers and organisations made nurses realise that they were not valued and therefore the need to protect their psychological wellbeing prompted their ‘job hopping’ action.

However, the nurses we spoke to did refer to being union members. Although collective action did not seem to be a conscious strategy for most nurses during COVID-19 many did feel able to contact their unions to seek clarification over issues. For example, Gaby contacted her union to get advice on redeployment at the start of the pandemic, passing-on the information she was given to worried colleagues:

I know that people that were being deployed. We used the union (…) I’m a workplace rep. So I was able to kind of get some advice about [redeployment] when people are very worried

**Gaby**, **interview time-point 1**

For many nurses, raising concerns with unions was done as an individual action and the union was able to provide information and advice. Therefore, rather than inspiring collective action during the COVID-19 pandemic, contact with their unions during the pandemic appeared to allay individual concerns. However, taking future political action was not ruled out by the nurses and many referred to it in their narratives. Most of the nurses we spoke to agreed that pay was a contentious issue and one that many stated they would be willing to potentially take political action (strike or action short of a strike action) over, in line with their union’s stance.

## Discussion

This paper took as a starting point nurses’ experiences during the COVID-19 pandemic in the UK with the traumatic and distressing working practices they experienced in altered care-landscapes with more critically ill patients. Our analysis of interviews at earlier stages of the pandemic identified systemic challenges, nurses’ experiences of moral distress, and psycho-social impacts to further explore the transitions and multiplicity of experiences encountered by the nurses across the trajectory of the pandemic. Analysis of longitudinal interviews have revealed the nurses’ use of agency across the COVID-19 pandemic, with nurses’ pandemic-trajectories characterised by ‘getting by’, getting out or job-hopping, getting their needs met and ‘getting organised’.

Although we have characterised the nurses’ trajectories as agentic, the constraints they experienced daily whilst working during the pandemic, impacted on their ability to take agentic action. Pandemic unpreparedness within the NHS, for example in regard to PPE supplies, and staff shortages, exacerbated by staff absences due to COVID-19, made a previously extremely difficult situation almost untenable for many nurses [8]. The organisational deficiencies of the NHS were accentuated during the COVID-19 pandemic and these difficulties were compounded by the ‘psychological vulnerability-stigma nexus’ which operated within a culture of invulnerability [36]. However, many nurses did use their agency to be able to cope with these constraints to continue their workings lives within nursing and therefore with their nursing identity in-tact. The pandemic accentuated the nurses tough working conditions and left the majority of nurses feeling undervalued and embittered. It was often the realisation of these emotions towards their employers that acted as a kind of critical [20, 22] or pivotal moment. These pivotal moments were visible in the data where nurses made a change to their pandemic-trajectories, for example in prioritising their own wellbeing or job hopping. There has been a recent trend amongst advocates of neo-liberal discourses to argue that structuring categories such as gender have given way to increasingly individualised biographical patterns shaped by choice and therefore individual agency [43]. However, we believe that in practice individual’s daily lives are shaped profoundly by structural and environmental factors. To understand what was different about the nurses who felt able to use their agency for example, to take a break, as compared to those who continued to ‘get by’, we turn again to Lister’s [26] ideas regarding the range of unequally distributed assets or resources as one factor in people’s differential ability to cope with stressful circumstances. In this paper we have aimed to give due attention to agency, but within the context of the barriers and obstacles nurses faced. The optimal environmental conditions had to exist to enable the nurses to use their own agency, and pivotal moments only seemed to occur prior to nurses using their own agency. In fact, pivotal moments seemed to be given, by nurses who experienced them, as a justification for the transitions and changes they made which profoundly affected their identities, such as the transition from being a nurse who worked in the NHS to one who worked in a private hospital or from being a nurse who did not take sick leave to one that did. We found that for those nurses who felt able to ‘get their needs met’ by prioritising their own wellbeing (or taking time off work) all of them (apart from Raheem) had supportive managers. When the environmental conditions were not optimal, for example, not having supportive managers, nurses felt the presence of the psychological vulnerability-stigma nexus loom large and appeared unable to take action, other than to continue to strategically enable themselves to ‘get by’, or as in Annabel’s case, job hop.

In their narratives, nurses who got by did not refer to pivotal moments. It was apparent from our data that nurses who worked in care homes, hospitals and community settings or had been redeployed were more likely to report the occurrence of pivotal moments and to have had trajectories which included ‘getting by’, ‘getting needs met’ or ‘getting out’. The distress and trauma frequently associated with redeployment, particularly for non-ICU nurses who were sent to work in ICU with minimal training, appears to be comparable to the experiences of many nurses who worked in hospital, community and care home settings [9]. In these settings nurses worked with patients who were critically ill physically, and where death frequently occurred. Therefore, as we argued in Conolly et al., [37] we believe the importance of the nurses’ working environment cannot be underestimated. There is a need for UK policy initiatives to focus improvements on environmental factors and systems, such as creating cultures of supportive management [3, 37]. For those patients who spoke out within their organisation, they often found that they were not heard by managers and organisations during COVID-19 and for some this resulted in ‘getting out’. This accords with recent literature which has explored how feeling ignored by managers and their organization can reduce nurses’ levels of commitment to their working role and increase the risk of stress, burnout and intention to leave [42]. In the context of the chronic healthcare underfunding in the UK [3], it is important to understand nurses COVID-19 trajectories and possible pivotal moments as encapsulating the key biographical themes certainly of their COVID-19 trajectories, if not of their working lives.

We would argue that the COVID-19 pandemic has forced a biographical disruption [22] due to nurses’ very altered working environments and increased the visibility of organisational disregard to nurses and their patients [9]. Nurses’ realisation of how their organisation (mis)treated them (or their inability to continue to ignore) and their inability to deliver patient care in line with their values and their own emotional response to this, lead some nurses to take agentic action in the form of leaving or taking a break from their working environment through either privileging their own wellbeing or job-hopping.

Notions of choice and fate permeate our longitudinal interview data, but in the context of nursing within the COVID-19 pandemic, it is important to remember that many of the structural deficiencies which have existed in the nursing profession for decades have been brought to the fore by the pandemic [6]. Giddens’ [22] model of the fateful moment does not provide an explanation of why particular courses of action are taken whilst others are not. As the sociology of transitions and poverty literature highlights, individual trajectories are subject to socio-structural constraints, but within this framework of factors and the impact of the macro-environmental there is space for individual agency [35]. Thus social, cultural and institutional factors can enhance or restrain autonomy [20]. Giele and Elder [44] argued that when undertaking longitudinal work, the four-factor model of the life course should be borne in mind. This includes time and place, linked lives, agency, timing and resourcefulness [44]. We agree with Holland and Thomson [43: 461] who argue that ‘critical’ or ‘pivotal’ moments are a central part of research participants ‘narratives of self’ and individuals’ ‘resourcefulness [that] they show in the face of such moments and the ways that such moments themselves are revisited, worked and reworked in the ongoing biographical narrative’ are equally important. Thus, the conceptualisation of biographical disruption is useful when considering the nurses narratives as this refers to the context of organisational and structural factors which were in place prior to the COVID-19 pandemic and were accentuated and came to the fore during the intensified working conditions during this time. The identification of the four main types of agency nurses used in their pandemic trajectories raises an important point about the support and care nurses need going forward in order to keep doing their roles. For some nurses their main aim was simply stability and strategically trying to ‘get by’ rather than change and advancement. Trajectories are subject to change and many of the nurses slipped between these categories over the course of the pandemic. A strength of our qualitative data was that it involved a large sample of nurses who were interviewed up to four times over the course of the COVID-19 pandemic.

## Conclusions

Our use of the concepts of pivotal moments and biographical disruption have helped us to understand the ways nurses used their agency in their pandemic-trajectories. Most of the nurses made it very clear that their pandemic-trajectories occurred within the ‘psychological vulnerability-stigma nexus’ and they used their agency to navigate its implications. For some of the nurses this nexus was reinforced by their managers who were not supportive. Other nurses did refer to having supportive line managers. This suggests that some of the difficulty nurses had in their organisations due to the psychological vulnerability-stigma nexus was due to non-supportive cultures and the responsibility for this lies at a management and organisational level. The nurses’ willingness to take unpaid leave, job hop (which may deplete the workforce of experience and skills) and take strike action over fair pay highlights that more difficulties lie ahead for the nursing profession if nurses’ views are not taken into consideration and / or seriously. We call for more considered systemic support to be delivered and consistently provided to nurses and other healthcare workers to ensure the future of nursing, good retention of all healthcare workers and the security of society. The avoidance of a mass exodus, and any further deepening of recent UK and global nursing workforce shortages, depends on getting the right support in place now.
